# Sinus or Fistula Following Abdominal Operations

**Published:** 1898-08

**Authors:** J. Garland Sherrill

**Affiliations:** Lecturer on Surgery and Demonstrator of Anatomy in the Hospital College of Medicine, etc., Louisville, Kentucky


### Southern Medical Record.

A MONTHLY JOURNAL OF MEDICINE AND SURGERY.

VOL. XXVIII. ATLANTA, GA., AUGUST, 1898. NO. 8.

### Original Articles.

### SINUS OR FISTULA FOLLOWING ABDOMINAL OPERATIONS.

By J. GARLAND SHERRILL, M. D.

Lecturer on Surgery and Demonstrator of Anatomy in the Hospital College of Medicine, etc., Louisville, Kentucky.

Stenographically reported and condensed for this Journal, by C. C. Mapes, Louisville, Ky.*

At a recent meeting of the Louisville Surgical Society the author read a paper upon this subject in which he said in part:

Perhaps no condition resulting from abdominal operations will be more persistent and more distressing (to the patient) than sinus or fistula, which are so closely allied as to practically come under the same heading.

It is well to divide sinuses into simple suppurating sinus, and fistula, which may be either fecal, urinary, or biliary. Simple sinus presents a tract lined by granulation, which extends into the abdominal wall or the abdominal cavity, frequently communicating with an abscess sac at the bottom of the pelvis. Fistula presents a similar condition with an opening into a viscus discharging its contents through the tract. Sinuses in the abdominal wall almost invariably heal under proper care, but repair of deeper sinuses is protracted, and recovery without operative measures the exception and not the rule. They may persist for years, the patient finally dying of exhaustion. Fecal fistulæ rarely close spontaneously, more often remain patulous. If the upper part of the intestine is involved the patient emaciates rapidly from lack of nourishment. The skin about a fistulous opening often becomes excoriated because of the discharge; considerable induration occurs around the tract due to development and contraction

*NOTE.—The original paper was published in Medicine, Vol. 4, No. 2. The discussion which follows has not before appeared in print.

of new connective tissue. As a rule the longer the fistulous tract the more troublesome is the condition. Biliary and vesical fistula frequently heal spontaneously with simple drainage.

Wounds which heal without suppuration seldom leave a fistula; this condition results from infection with a suppurative focus which discharges in the line of incision. The tract remains patulous usually because there is an infected ligature at the bottom; if this comes away the tract may heal. The opening may close, and subsequently reopen because the pus sac below is still secreting. Healing may be prevented by adhesions. Openings into the intestine, bladder or gall-bladder act as a source of irritation and prevent healing. Sometimes the surgeon has intentionally left an artificial opening to be closed later. I am convinced that an operation should be completed, even if resection be necessary, rather than leave a fistula. Shock is not markedly increased, and danger to life is less than that entailed by a later operation for an exhausting fistula. I do not include cases which demand a permanent fecal fistula.

"The formation of fecal fistulæ by fixing the open ends of the gut in the abdominal wound is a cruel proceeding, unless it be absolutely impossible to treat the case in any other way. If the patient recovers he lives in a more or less miserable condition and has usually to look forward to a future operation." (Keith.)

Injuries to the intestine which are overlooked will invariably result in fistulæ. Drainage, especially by gauze, may act as a cause. Gauze becomes agglutinated to the tissues and slight force necessary to remove it may tear a weakened bowel. Drainage tubes and large silk sutures imperfectly sterilized may cause fistulæ.

"The drainage tube is no doubt accountable for the formation of some simple fistulæ, but thick, knotted silk probably accounts for most. Pressure necrosis by forceps or drainage tube may produce fecal fistulæ, and severe traumatism, or simple tearing might have the same effect. These are causes which are not easily avoided, although they should be guarded against; but the production of fistulæ through the employment of silk ligatures which are either too thick or too long or insufficiently embedded can and ought to be avoided." (Greig Smith).

The treatment should be: First, preventive; second, curative. Preventive treatment embraces precaution against sepsis, care during operation to prevent injury of a hollow viscus; when such injury occurs, complete closure of the rent; drainage to be used only when absolutely indicated, a glass drain being preferred. Gauze may be used when oozing demands it as a pack, but as a drain it is not effective, and great care should be exercised in its removal. The smallest silk consistent with safety should be employed for litigation of pedicles; before it is applied the wound should be freshly draped with gauze; in suppurative cases the ligature should be kept from contact with pus.

When sinus has developed several plans of treatment offer themselves: First, packing daily with gauze, either firmly or a small wick carried to the bottom; second, a rubber or glass drain; third, injections into the tract; fourth, operative measures, curettement or radical. Whatever plan of palliation is selected, cleanliness is of primary importance; the part should be dressed once daily, or oftener if there is much discharge. In simple sinus the bowels should be kept open. It is better to avoid purgatives in fecal fistulæ, and depend upon enemata to evacuate the bowel unless the fistula communicates with the lower part of the rectum.

Simple sinuses may be palliated for a considerable time hoping for discharge of the infected ligature. It may be found advisable to use some stimulating injection with the hope that repair of the tract may occur, which, however, will be seldom realized. After several months, more radical measures must be considered. If the sinus is not deep, curetting will sometimes prove curative; if the tract is long and tortuous, or there is an intestinal or vesical communication, little good will be accomplished. Then the abdomen should be reopened above the tract, after cleansing by irrigation, and separating adhesions between the intestines, the ligature searched for and removed; after which the whole fistulous tract should be opened and the abdomen thoroughly cleansed and closed. This will likely prove a difficult undertaking and one of no little gravity. No effort should be made to enlarge the orifice of the sinus, as there is danger of opening an intestine, and the absence of landmarks will be confusing.

In fecal fistulæ it is more difficult to determine when operation shall be performed. Danger of peritonitis following entrance of fecal matter into the peritoneal cavity will demand that sufficient time elapse for walling off the general cavity before operative steps are attempted. Such delay should be advised if the patient's condition will permit, but if rapidly emaciating a better chance for life is offered by immediate attempt to close the intestine, after emptying the fistulous tract. The method for closure of the gut must be determined in each individual case. In some the clamp can be used with success; in others resection with end-to-end suture will be advisable; while in others the simple approximation of the pared edges will restore the continuity of the intestine. None of the devices for re-establishing the continuity of the intestine without opening the abdomen have proved successful. The tract of fistulæ in the abnominal wall must, after adhesions are removed, be dissected out and approximated.

